# Embodied Intelligence Applications in Health Care Populations: Scoping Review

**DOI:** 10.2196/83871

**Published:** 2026-06-12

**Authors:** Wan Shu, Ying Zhou, Erhong Sun, Lei Deng, Xuchun Ye

**Affiliations:** 1 School of Nursing, Naval Medical University, No. 800, Xiangyin Road, Shanghai, 200433, China, 86 13336088171

**Keywords:** embodied intelligence, artificial intelligence, health care, scoping review, robotics

## Abstract

**Background:**

Embodied intelligence—artificial intelligence instantiated in physical or virtual bodies that can perceive, communicate, and interact with users and their environments—has been increasingly applied in health care. However, the evidence base remains fragmented because of inconsistent terminology, diverse embodiment forms, and limited synthesis of application domains, target populations, care settings, acceptability, and effectiveness. This fragmentation constrains conceptual clarity and translation into routine health care practice.

**Objective:**

This scoping review aimed to systematically map the applications of embodied intelligence in health care by classifying embodiment forms, identifying major functional domains, describing target populations and implementation settings, and synthesizing the available evidence on acceptability and effectiveness.

**Methods:**

This scoping review followed the Arksey and O’Malley framework, with enhancements by Levac et al, and was reported in accordance with the PRISMA-ScR (Preferred Reporting Items for Systematic Reviews and Meta-Analyses extension for Scoping Reviews) and PRISMA-S (Preferred Reporting Items for Systematic Reviews and Meta-Analyses literature search extension) guidelines. Seven electronic databases were searched from database inception to December 2025, supplemented by gray literature searches and backward citation screening. Eligible studies were primary empirical studies published in English or Chinese that examined embodied intelligence in health care contexts. Two reviewers independently screened records and charted data using a pilot-tested standardized form. Descriptive statistics and thematic synthesis were applied. No formal critical appraisal was conducted because the aim was to map the breadth and characteristics of the evidence base.

**Results:**

A total of 83 studies were included. Five embodiment forms were identified: virtual humanoid agents (32/83, 38.6%), physical humanoid robots (32/83, 38.6%), virtual animal-shaped agents (1/83, 1.2%), physical animal robots (13/83, 15.7%), and mechanical robots (5/83, 6%). Applications clustered into 3 functional domains: health management and health education (40/83, 48.2%), mental health promotion (37/83, 44.6%), and physiological health promotion (6/83, 7.2%). Older adults were the most frequently targeted population (45/83, 54.3%). Interventions were mainly implemented in home settings, care homes, laboratories, and hospitals. Twenty-two randomized controlled trials reported generally beneficial effects on health behaviors, mental health outcomes, or cognitive function, although outcome measures were heterogeneous. Twelve studies examined acceptability and generally reported favorable user acceptance.

**Conclusions:**

This scoping review provides the first comprehensive synthesis of embodied intelligence in health care using a unified classification of forms, functional domains, populations, and application settings. The findings indicate that embodied intelligence is most mature in “health management and health education” and “mental health promotion,” with increasing real-world deployment in home and care home settings. By consolidating fragmented evidence and standardizing terminology, this review offers a practical foundation for clinicians, nurses, and policymakers to support the implementation of embodied intelligence in routine health care. Evidence is limited by heterogeneous outcome measures, many lab-based evaluations, and the absence of formal quality appraisal, underscoring the need for standardized outcome measures, rigorous randomized controlled trials, and longitudinal evaluations to enable scalable and ethically grounded real-world adoption.

## Introduction

### What Is Already Known

Embodied intelligence has been piloted in health care, but prior research remains fragmented and lacks a systematic classification of embodiment forms, functional roles, and application contexts. Small-scale studies suggest that embodied intelligence may support rehabilitation and provide companionship; however, there is still no comprehensive map of where, how, and for whom these systems have been implemented.

### What This Paper Adds

We systematically classified embodied intelligence in health care into 5 embodiment forms and 3 core functional domains. By synthesizing evidence from 22 randomized controlled trials (RCTs) and 12 mixed methods acceptability studies, we summarized the reported effectiveness of embodied intelligence in health management and health education and mental health promotion, as well as patterns of user acceptability across settings. These findings provide an evidence base to inform future standardized evaluation and implementation of embodied intelligence in health care, with particular relevance to nursing practice.

### Rationale

As global populations age and chronic diseases become increasingly prevalent, health care systems face unprecedented challenges [1,2]. Conventional care models often struggle to address diverse and individualized patient needs, prompting growing interest in artificial intelligence (AI)–enabled solutions in health care [3]. In recent years, AI has been widely applied to tasks such as diagnosis, risk prediction, and clinical decision support, contributing to improvements in efficiency and accuracy across health care systems [4,5].

Embodied intelligence represents a distinct branch of AI that integrates computational intelligence with physical or virtual embodied entities, enabling direct interaction with users and their environments [6]. In this review, embodied intelligence refers to AI systems instantiated in physical or virtual forms that interact with users through perception, communication, and action, including virtual humanoid agents, physical humanoid robots, animal-shaped agents, and mechanical robots applied in health care contexts.

In this context, embodiment refers to the integration of intelligence with a physical or virtual form that enables situated perception, action, and interaction within an environment [7]. In this review, embodied intelligence is used as an umbrella term for embodied AI systems that exhibit interactive and adaptive behaviors through their physical or virtual embodiment. Embodied robots represent a subset of embodied intelligence, referring specifically to physically instantiated robotic systems with intelligent capabilities, whereas virtual embodied agents (eg, virtual humanoid or animal-shaped agents) represent nonphysical yet embodied forms of intelligent systems. To avoid ambiguity, this review adopts embodied intelligence as the overarching term and classifies systems based on embodiment form rather than using related terms interchangeably.

Although AI has been increasingly integrated into health care, many widely deployed AI applications remain disembodied, functioning primarily as background analytical tools such as diagnostic algorithms, risk prediction models, or data-driven decision support systems [8]. These systems typically operate at the level of information processing and decision-making, with limited direct interaction with patients or clinicians.

In contrast, embodied intelligence emphasizes embodiment as a core design principle, integrating intelligence not only algorithmically but also through physical or virtual presence, environmental context, and interactive engagement. By enabling multimodal interaction—such as dialogue, movement, facial expressions, or touch—embodied intelligence can extend AI from passive analytical support toward more active participation in care processes. This interactive and socially situated nature makes embodied intelligence particularly relevant for health care domains that rely on sustained engagement, trust, and relational interaction, including health education, chronic disease management, mental health support, and nursing care [6]. Despite growing interest in embodied intelligence for health care applications, existing studies remain fragmented with respect to embodiment types, functional capacities, and the evaluation of effectiveness and acceptability.

Several recent reviews have examined AI and robotics in health care from specific perspectives. For example, Fiske et al [9] explored the ethical and societal implications of embodied AI in mental health–related fields such as psychiatry, psychology, and psychotherapy. Huang et al [10] conducted a systematic review of intelligent physical robots in health care, focusing on antecedents, consequences, and organizational factors associated with robot implementation. Kiuchi et al [11] provided a meta-review emphasizing psychological insights into embodied conversational agents, chatbots, and social assistive robots, with particular attention to interaction design and user experience.

While these reviews offer valuable insights, they primarily concentrate on specific embodiment forms, disciplinary viewpoints, or outcome domains. In contrast, this scoping review adopts a broader and integrative perspective by examining multiple types of embodied intelligence—both physical and virtual—across diverse health care contexts. By systematically mapping embodiment forms, functional roles, application settings, and evidence of acceptability and effectiveness, this review aims to clarify the current landscape of embodied intelligence in health care and identify priority research gaps to inform future research and practice.

Despite the growing body of research on embodied intelligence in health care, there remains a lack of integrative evidence that systematically examines how different forms of embodied intelligence are applied across health care contexts, what functional roles they serve, and how acceptability and effectiveness are evaluated [12]. Existing studies are often fragmented by technology type, application domain, or outcome focus, making it difficult to obtain a comprehensive understanding of the field.

### Objectives

To address these gaps, this scoping review synthesizes applications of embodied intelligence in health care by mapping embodiment forms, functional domains, application settings, and evidence of acceptability and effectiveness. By providing an integrated overview of current practices and identifying priority research gaps, this review aims to inform future research and support the responsible integration of embodied intelligence into health care and nursing practice.

Accordingly, this scoping review addresses the following research questions:

1. What types of embodied intelligence have been applied in health care, and how are they classified based on their embodiment forms?
2. What functional roles do different types of embodied intelligence serve in health care applications?
3. In which health care contexts and settings have embodied intelligence systems been implemented?
4. What evidence has been reported regarding the acceptability and effectiveness of embodied intelligence in health care?

## Methods

### Protocol and Registration

A scoping review was conducted according to the framework described by Arksey and O’Malley [13] with an extended version by Levac et al [14]. This review is reported based on the PRISMA-ScR (Preferred Reporting Items for Systematic Reviews and Meta-Analyses Extension for Scoping Reviews) guidelines (Checklist 1) [15]. The protocol was registered on the Open Science Framework (OSF) [16]. The review methods were implemented before OSF registration and were subsequently documented in the registered protocol. The review title and wording were refined during manuscript development to better reflect the scope of the evidence map; however, no substantive changes were made to the eligibility criteria, information sources, screening procedures, or synthesis approach.

### Eligibility Criteria

The eligibility criteria were developed using the PCC (population, concept, and context) framework [17] to ensure alignment with the review objectives. The population of interest included any human participants, such as patients or healthy individuals, who interacted with or were affected by embodied intelligence systems in a health care context. Specific population characteristics, including age and health status, were not used as eligibility criteria but were charted during data extraction.

The core concept was embodied intelligence, defined in this review as AI systems integrated with a physical or virtual form capable of interacting with users and/or the environment through situated perception, communication, or action. Eligible systems included, for example, virtual embodied conversational agents, humanoid robots, animal-shaped robots, and mechanical robots. Embodied intelligent robotics was considered a subset of embodied intelligence referring specifically to physically instantiated robotic systems with intelligent and interactive capabilities; the two terms were not used interchangeably.

The context included any health care–related setting, such as hospitals, clinics, long-term care facilities, residential settings, research laboratories, and community-based programs.

Primary empirical studies of any design were eligible for inclusion, including RCTs, pilot studies, quasi-experiments, observational studies, qualitative studies, mixed methods studies, case reports, and crossover trials. Studies published in English or Chinese were included. Studies were excluded if they were nonprimary research, such as reviews, editorials, letters, or study protocols, or if they were not available as full peer-reviewed journal articles with sufficient methodological detail for screening and data charting, such as conference abstracts without full papers, dissertations, theses, or other gray literature items.

For study selection, embodiment was operationally defined as the presence of a physical or virtual form that enabled situated interaction with users and/or the environment, such as visual representation, movement or gesture, facial expressions, voice interaction, or tactile interaction.

### Information Sources

A comprehensive search was conducted in 7 electronic databases: PubMed (National Library of Medicine interface), Web of Science Core Collection (Web of Science platform), Cochrane Library (Wiley platform), MEDLINE (via Ovid), APA PsycNet (APA PsycNet platform), CQVIP (CQVIP platform), and China National Knowledge Infrastructure (CNKI) (CNKI platform). No separate search update was undertaken beyond the final searches completed in December 2025. All databases were searched from database inception to December 2025. No simultaneous multidatabase searching on a single platform was undertaken.

All retrieved records were imported into EndNote (Clarivate) for deduplication. Duplicate records were removed using automated matching followed by manual verification. The most recent search was conducted in December 2025.

### Search Strategy

A 3-step search strategy was applied to identify relevant studies from database inception to December 2025, with search reporting guided by the PRISMA-S (Preferred Reporting Items for Systematic Reviews and Meta-Analyses literature search extension) guideline (Checklist 2) [18]. First, 7 electronic databases were searched. The search strategy was adapted for each database and is presented in full (line by line) in Multimedia Appendix 1.

Second, purposeful searching of online resources for gray literature was conducted using OpenGrey and Google Scholar. Third, backward citation searching was performed by screening the reference lists of included studies and relevant reviews to identify additional potentially eligible records.

No date limits were applied at the database search stage, and all databases were searched from inception to December 2025. Language restrictions were limited to English and Chinese to match the review scope and the language capacity of the review team. Eligibility restrictions to primary research studies reporting original empirical data were implemented during study selection. No published search filters were used. The search strategies were developed for this review and were not directly adapted from previous literature reviews. No study registries were searched as part of this review. No additional studies or data were sought by contacting authors, experts, manufacturers, or other stakeholders. The PRESS (Peer Review of Electronic Search Strategies) guideline was not conducted and is acknowledged as a limitation.

### Selection of Sources of Evidence

Study selection was conducted in two stages by two independent reviewers. In the first stage, titles and abstracts were screened against the eligibility criteria. In the second stage, the full texts of potentially eligible studies were retrieved and assessed for final inclusion.

Any discrepancies between reviewers at either stage were resolved through discussion and consensus. All screening decisions were documented using a standardized screening protocol to ensure consistency and transparency throughout the selection process.

### Data Charting Process

Data charting was conducted using a standardized data extraction form that was developed and pilot-tested prior to formal extraction. Two reviewers independently extracted data from all included studies, and any discrepancies were resolved through discussion until consensus was reached.

To support consistency and traceability, all coding decisions and charting procedures were documented in a shared Microsoft Excel file. Chinese-language information extracted from included studies was translated into English by one reviewer and cross-checked by another reviewer before synthesis.

### Data Items

The data charted from the included studies included the authors, year of publication, country or region where the study was conducted, study design, study population or target group, type of embodied intelligence, functional role, application setting, and key outcomes.

These variables were selected to capture both the technological characteristics of embodied intelligence, such as embodiment form and role, and the application-oriented characteristics relevant to interpretation, such as population, setting, and outcomes. For the purposes of charting and synthesis, embodied intelligence was classified along 2 dimensions: material instantiation, defined as virtual versus physical, and morphology, defined as humanoid, animal-shaped, or mechanical. Functional domains were classified inductively according to the primary aim reported in each study.

When studies reported overlapping objectives, classification was based on the dominant aim described by the authors and the primary outcomes emphasized in the study. These operational decisions were made to facilitate consistent comparison across heterogeneous sources of evidence.

### Critical Appraisal of Individual Sources of Evidence

A formal critical appraisal of individual sources of evidence was not conducted. This review was designed as a scoping review to map the breadth, characteristics, and application patterns of the available evidence rather than to estimate pooled effects or exclude studies based on methodological quality.

Given the heterogeneity of study designs and the exploratory nature of the field, all eligible primary studies were retained for charting and narrative synthesis. The absence of formal critical appraisal is acknowledged as a limitation and is considered when interpreting the findings, particularly those related to acceptability and effectiveness.

### Synthesis of Results

To facilitate the synthesis and analysis of the included studies, a standardized data extraction form was developed, and the application of embodied intelligence and the roles played in health care were then grouped thematically through an inductive approach. This process involved two independent reviewers (WS and YZ) who independently read through the full text of each study to assign initial codes to relevant segments describing the technology’s use, function, or context. These initial codes were subsequently organized into broader categories or themes; for example, codes related to health education, disease self-management, and medication adherence were grouped under the overarching theme of “health management and education,” while codes concerning emotional support, cognitive stimulation, and reduction of loneliness were clustered under “mental health promotion.” The reviewers met regularly to discuss their coding and theme development, resolving any discrepancies through consensus. All coding decisions and theme definitions were documented in a shared coding sheet (Microsoft Excel) to support consistency and traceability; no qualitative analysis software was used. Additionally, countries, participants, settings, and study designs were mapped, and trends in publication numbers were analyzed to identify patterns and gaps in the existing literature. Mapping outputs (including evidence distribution across embodiment forms, functional domains, and settings) were generated through descriptive frequency summaries and cross-tabulation.

## Results

### Selection of Sources of Evidence

**Figure 1.**
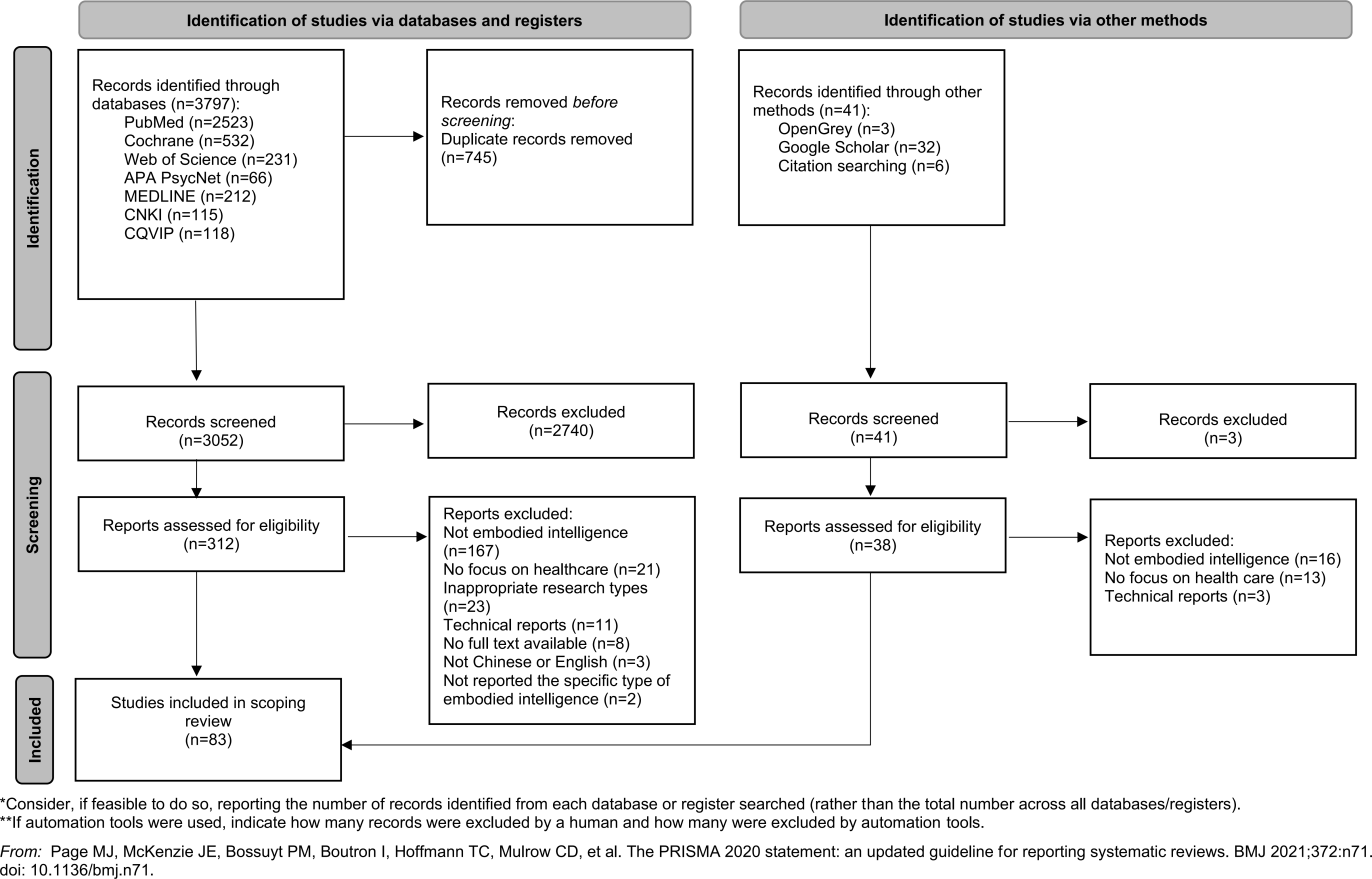
PRISMA (Preferred Reporting Items for Systematic Reviews and Meta-Analyses) 2020 flow diagram illustrating the study selection process for a scoping review on embodied intelligence in health care. CNKI: China National Knowledge Infrastructure; CQVIP: VIP Chinese Journal Database.

The detailed study selection process is illustrated in Figure 1. A total of 3797 records were initially identified through electronic database searches, and an additional 41 records were identified via other methods, including gray literature and citation searching, bringing the total to 3838 records. After removing 745 duplicates, 3093 records remained for screening. Of these, 2743 records (2740 from databases and 3 from other methods) were excluded during title and abstract screening, leaving 350 records for full-text assessment. At the title/abstract stage, records were excluded if they clearly did not meet the PCC-based eligibility criteria (eg, not involving embodied intelligence as defined in this review, not in a health care context, not primary research, not involving human participants, or not published in English/Chinese). Following a comprehensive evaluation of the full texts, 267 records were excluded (235 from databases and 32 from other methods) for reasons such as not focusing on embodied intelligence or health care, inappropriate study designs, or lack of available full text. This resulted in a final total of 83 studies included in this scoping review.

### Characteristics of Sources of Evidence

**Figure 2.**
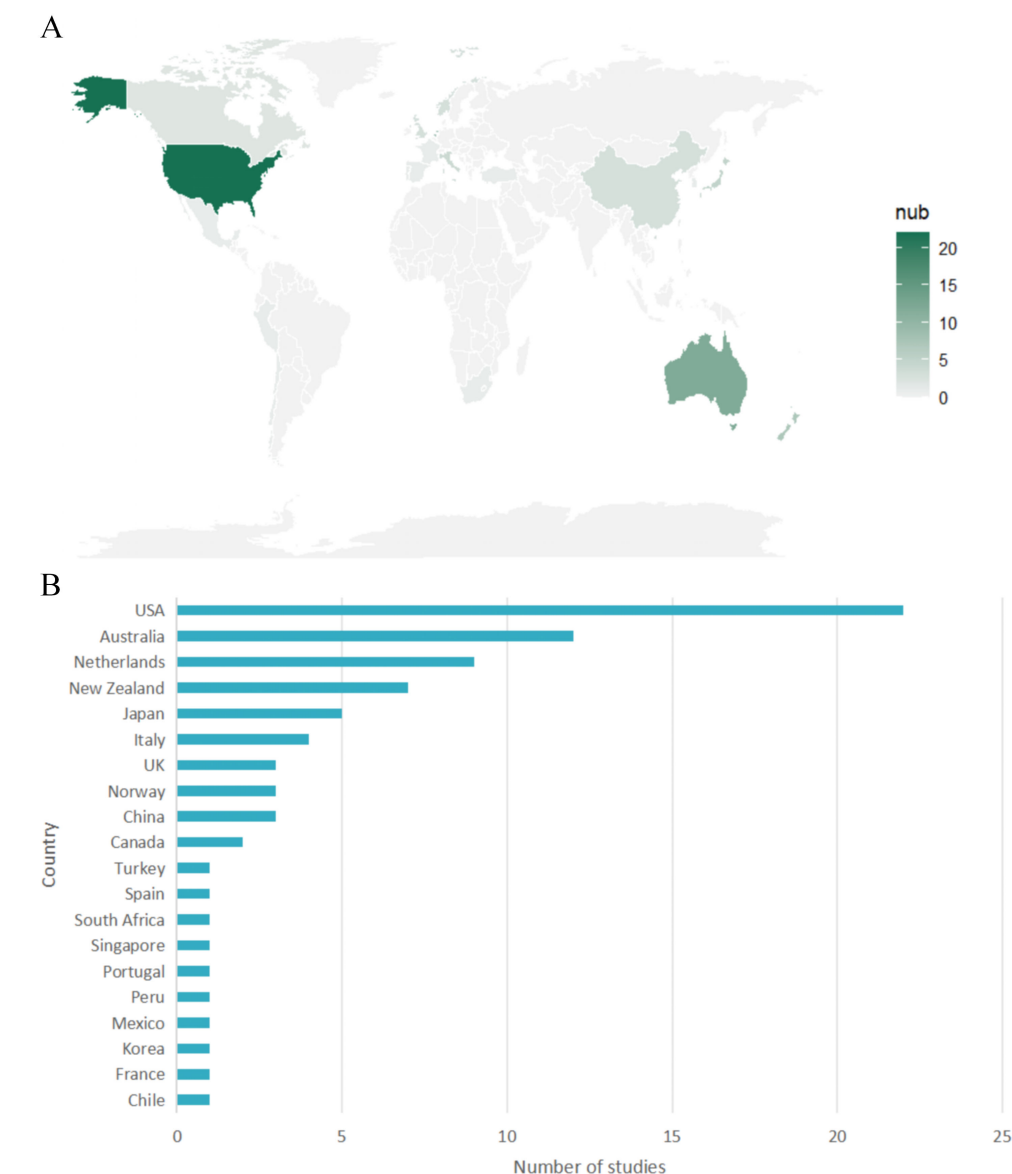
Characteristics of included studies in the scoping review. Panel A shows the geographic distribution of the 83 included studies by study setting (country/region where the study was conducted). Panel B presents the trends in publication volume by year and publication period.

There were 83 eligible studies published in English or Chinese according to the inclusion criteria. They were conducted in 20 countries, with more than half of the studies originating from Europe and the United States (Figure 2A and Table 1). Overall, the number of publications increased over time, peaking in 2021, followed by a marked decline in 2022 (Figure 2B). Study designs were diverse, with RCTs accounting for the largest proportion (nearly one-third) of all studies (Table 2). The characteristics of the included studies are presented in Multimedia Appendix 2 [18-99].

**Table 1.** Geographic distribution of the 83 included studies by study setting.

Country	Included studies, n
United States	22
Australia	12
Netherlands	9
New Zealand	7
Japan	5
China	4
Italy	4
Norway	3
United Kingdom	3
Canada	2
Spain	2
Chile	1
France	1
Korea	1
Mexico	1
Peru	1
Portugal	1
Singapore	1
South Africa	1
South Korea	1
Turkey	1

**Table 2.** The distribution of study designs.

Study design	Count, n
Randomized controlled trials	22
Pilot study	18
Quasi-experiments	15
Mixed methods studies	11
Qualitative study	7
Case studies	3
Crossover trials	2
3×3 factorial design	1
Descriptive observational pilot study	1
Observational cohort study	1
Pre-post study	1
Quasi-experimental research design	1

**Figure 3.**
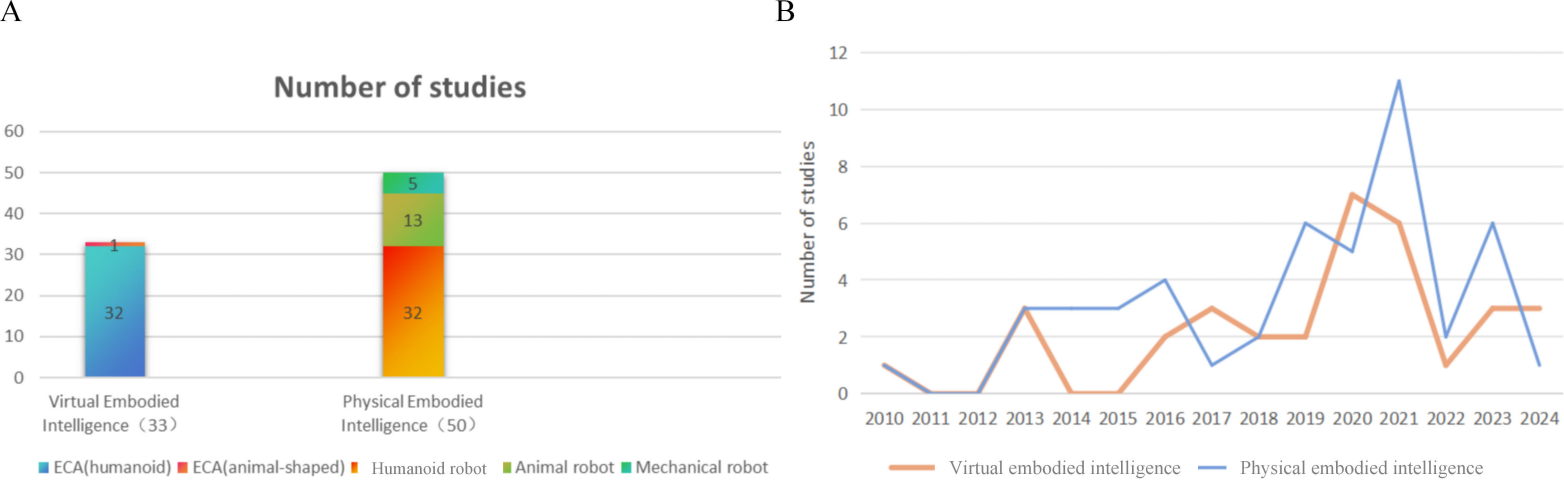
Categories and publication trends of embodied intelligence applied in health care. Panel A displays the distribution of embodied intelligence categories (virtual vs physical, including humanoid, animal-shaped, and mechanical forms) across the 83 included studies. Panel B illustrates temporal trends in publications comparing virtual embodied intelligence and physical embodied intelligence. The data derive from studies conducted in multiple countries and health care settings between database inception and December 2025.

A review of the literature indicates that embodied intelligence can be categorized into virtual embodied intelligence and physical embodied intelligence based on whether the system has a physical form. Virtual embodied intelligence is predominantly composed of human-shaped embodied conversational agents (32/33, 96.9%), whereas within physical embodied intelligence, human-shaped robots account for 64% (32/50), followed by animal-shaped robots (13/50, 26%) and mechanical robots (5/50, 10%) (Figure 3A). Physical embodied intelligence represents the majority of health care applications (50/83, 66.2%). As shown in the publication trend graph (Figure 3B), over the past 5 years, publications on physical embodied intelligence have generally exceeded those on virtual embodied intelligence.

**Figure 4.**
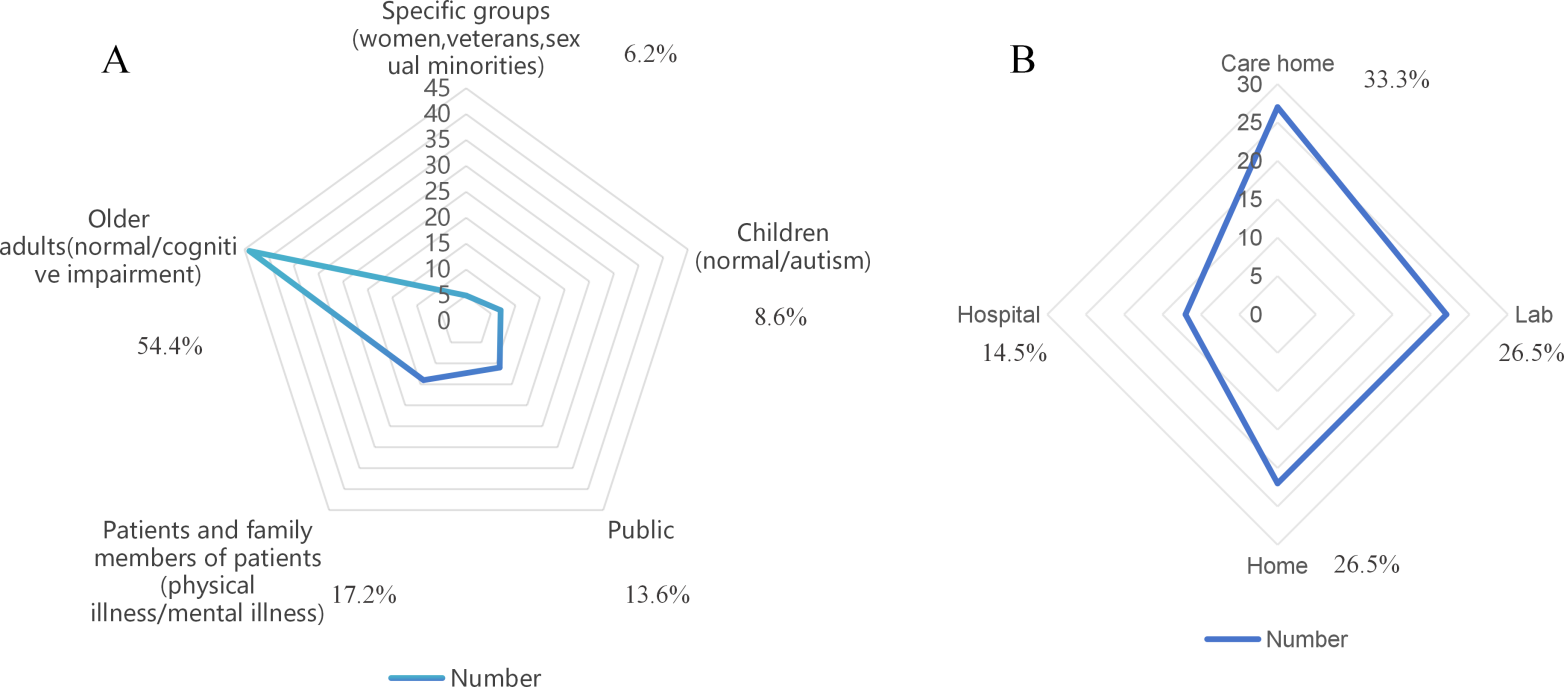
Study populations and application settings of embodied intelligence in health care. Panel A shows the distribution of target populations across included studies (eg, older adults, patients with chronic conditions, and children). Panel B summarizes the health care application settings, including home, care home, hospital, and laboratory environments. The figure reflects findings from 83 studies published between database inception and December 2025.

This review indicates that, although embodied intelligence has been applied across a range of health care scenarios, it is predominantly studied in older adults, who account for 54.4% (46/83) of the target populations (Figure 4A). Among these studies involving older adults, 69.6% (32/46) focused on healthy older adults and 30.4% (14/46) on individuals with cognitive impairment. This pattern likely reflects the increasing demand for elderly care services associated with rapid population aging. Implementation settings spanned 4 categories (Figure 4B): domestic residences (22/83, 26.5%), long-term care facilities (27/83, 32.5%), laboratory-based evaluation contexts (22/83, 26.5%), and hospital units (12/83, 14.5%). In this review, “laboratory” refers to controlled research environments (eg, university or clinical research labs) used primarily for prototyping and evaluation (eg, usability, interaction quality, feasibility testing, or preliminary outcomes), rather than real-world care delivery settings. While 73.5% (61/83) of studies were conducted in real-world care settings, over a quarter (22/83, 26.5%) were confined to controlled laboratory environments, which may limit ecological validity and the generalizability of findings to routine practice.

### Critical Appraisal Within Sources of Evidence

No critical appraisal within sources of evidence is reported because a formal critical appraisal of included studies was not undertaken in this scoping review. Consistent with the review aim of mapping the breadth and characteristics of the evidence base, all eligible studies were retained for data charting and synthesis regardless of methodological quality.

### Results of Individual Sources of Evidence

#### The Three Major Roles of Embodied Intelligence Applied in Health Care

Analysis of the 83 included studies indicates that embodied intelligence in health care serves 3 principal functional domains: health management and health education, mental health promotion, and physiological health promotion. In this review, the distinction between “health management” and “health promotion” is based on the primary intent of the intervention. Health management denotes structured, ongoing support aimed at controlling a specific disease, condition, or modifiable risk factor, typically through monitoring, symptom tracking, medication or lifestyle adherence support, and self-management guidance. Health education denotes interventions primarily intended to improve health-related knowledge, skills, and decision-making (eg, patient education or training), and was grouped with health management when education served a disease- or risk-focused management goal. In contrast, health promotion denotes interventions primarily intended to enhance well-being, function, or health capacity (eg, emotional well-being, cognitive engagement, physical function, or rehabilitation outcomes) without being anchored to the management of a single disease alone.

These categories were derived inductively during thematic analysis and were not predefined. Specifically, interventions centered on disease self-management, adherence, and condition-focused education were grouped under “health management and health education”; those centered on emotional support, anxiety reduction, or cognitive engagement were grouped under “mental health promotion”; and those centered on improving physical activity, functional performance, or rehabilitation outcomes were grouped under “physiological health promotion.” When studies reported overlapping objectives (eg, monitoring functions embedded within a rehabilitation program), classification was determined by the dominant aim described by the authors and the primary outcomes emphasized.

As summarized in Table 3, these functions were operationalized through 46 distinct embodied intelligence devices. Health management and health education (40/83, 48.2%) and mental health promotion (37/83, 44.6%) accounted for most applications. Most studies reported beneficial outcomes within their respective domains; 2 experimental studies did not report improvements and are marked (※) in Table 3.

**Table 3.** Roles of embodied intelligence in health care.

Types of embodied intelligence	Role		
	Health management and health education	Mental health promotion	Rehabilitation training
Virtual embodied intelligence (animal-shaped; n=1)		✓	
Virtual embodied intelligence (humanoid-shaped; n=25)	✓※^a^	✓	
Humanoid robot (n=12)	✓	✓	✓
Animal robot (n=2)	✓	✓※^a^	
Mechanical robot (n=6)	✓	✓	

a ※ indicates studies that did not report beneficial effects consistent with the stated role.

#### Health Management and Health Education

Among the 83 included studies, 40 examined the application of embodied intelligence in health management and health education.

First, in 30 studies, embodied intelligence served health management functions, covering both professional health management and self-management. Professional health management was mainly applied in medical services, experimental research, and other specialized scenarios. Specifically, these systems supported health data collection, disease screening, and assessments such as attention level evaluation in populations including people with diabetes or chronic obstructive pulmonary disease, pregnant women, and older adults. Based on these assessments, the systems delivered targeted and personalized management advice [18-41]. Reported roles included assisting clinicians with documentation, reducing clinical risks, and improving patients’ adherence to recommendations. Notably, ter Stal et al [40] quasi-experimental study reported low adherence, which was attributed to insufficient personalization and limited information accuracy.

In contrast, self-management functions were mainly implemented in home or nursing home settings to support recipients (eg, older adults or people with diabetes) with exercise guidance, smoking cessation support, and disease self-management outside clinical encounters [42-47]. These applications primarily aimed to improve individuals’ quality of life.

Furthermore, 10 studies focused on the application of embodied intelligence in health education. Researchers integrated educational content into embodied intelligence systems to deliver health education to diverse groups, including individuals with chronic conditions, people with alcohol use problems, older adults, and the general public. The findings suggest that embodied intelligence may improve participants’ health literacy. Compared with traditional education delivered by health professionals, embodied intelligence offered greater flexibility and convenience, mainly through reduced time and location constraints and potentially lower learning burden. Consequently, participants reported higher satisfaction and better acceptance of education delivered via embodied intelligence systems [48-57].

#### Mental Health Promotion

There were 37 studies focusing on the application of embodied intelligence in mental health promotion, addressing 3 main tasks: psychological intervention, cognitive enhancement, and emotional companionship.

##### Psychological Intervention

In recent years, embodied intelligence has attracted increasing attention as a delivery modality for psychological interventions. Of the 37 studies, 6 integrated cognitive behavioral therapy and/or mindfulness-based practices into embodied intelligence systems. Findings suggest that these interventions may enhance subjective well-being and reduce distress among university students and members of the general public with psychological difficulties [12,58-62].

##### Cognitive Enhancement

Embodied intelligence–supported cognitive interventions have also been explored as a nonpharmacological approach. Among the included studies, 3 focused on using embodied intelligence to deliver cognitive training for older adults [63-65]. These studies reported that cognitive stimulation games and other embodied intelligence–based training may improve memory and executive function, while also promoting engagement in activities and social interaction. For example, Park et al [66] implemented a 6-week embodied intelligence–assisted multimodal cognitive training program and observed significant improvements in indicators such as memory and executive function, supporting the potential of embodied intelligence to enhance cognitive functioning in older adults.

##### Emotional Companionship

A total of 28 studies examined embodied intelligence for emotional companionship. Of these, 22 (78.6%) targeted older adults. The findings suggest that embodied intelligence can provide emotional support through multimodal interactions (eg, speech, facial expressions, and movement) and that older adults are generally receptive to embodied intelligence in a companion role. Reported benefits can be summarized at 3 levels.

First, at the psychological level, emotional bonds formed between embodied intelligence and older adults were associated with reduced depressive symptoms, loneliness, and psychological distress, and may even modulate perceived pain [24,58,66-76].

Second, at the social level, embodied intelligence was reported to facilitate social participation and improve social integration among older adults [77-83].

Third, at the family level, embodied intelligence may act as a shared social medium that supports intergenerational communication and strengthens family interactions through shared use [84,85]. Overall, embodied intelligence may elicit positive emotional experiences in older adults, which can motivate them to share these experiences with others and potentially increase opportunities for social engagement. As a novel technology, embodied intelligence may also function as a social catalyst and conversational topic in peer interactions.

Beyond older adult populations, 6 studies involved children or individuals with depression. Four studies focused on pediatric populations; 3 reported that embodied intelligence could reduce anxiety and enhance social interaction by engaging children (including those with autism) through voice-based interaction [86-88]. However, 1 study reported different findings. Kitt et al [89] quasi-experimental evaluation of an animal-like robot (Paro) in pediatric health care settings found no stress-reducing effect. This discrepancy may reflect differences in role framing and study design: in Crossman et al [87], the robot provided comforting interaction after children completed a stressful task, whereas in Kitt et al [89], the robot was involved during the stressful task itself, which may have been perceived as disruptive rather than supportive.

Two studies involving individuals with depression suggested that embodied intelligence, through verbal interaction tailored to users’ emotional states and needs, may provide emotional companionship and reduce perceived social stigma and internalized self-stigma [89,90].

### Physiological Health Promotion

Embodied intelligence was applied for physiological health promotion in 6 included studies, covering sensory training for children with autism and motor rehabilitation for individuals with mobility impairments; however, most of these studies (5/6) were conducted in laboratory settings.

Three laboratory-based studies involving children with autism embedded traditional sensory training into embodied intelligence systems and used multimodal interaction (eg, voice and tactile interaction). These studies reported improvements in auditory comprehension, emotional comprehension, and attention [91-93].

In addition, for patients requiring exercise rehabilitation after cerebrovascular events or heart disease, embodied intelligence was used to monitor exercise performance in real time and provide timely feedback, helping patients track training effects and progress [94-96]. One study reported that patients’ acceptance of embodied intelligence–assisted rehabilitation was higher than that of traditional exercise-based rehabilitation programs [97].

### Evidence Distribution and Gaps Across Embodied Intelligence Types and Functional Domains

**Figure 5.**
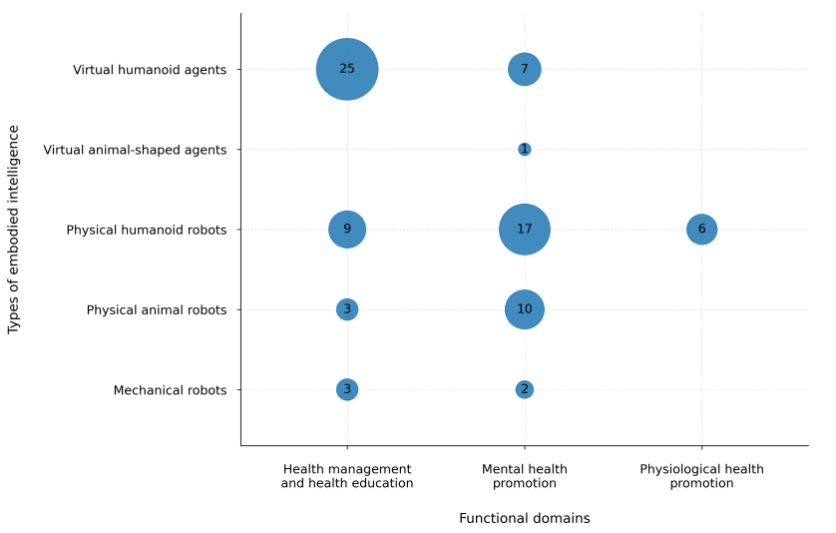
Bubble evidence gap map of embodied intelligence applications in health care.

As shown in Figure 5, the evidence base was unevenly distributed across embodied intelligence types and functional domains. Bubble size and the number displayed within each bubble represent the number of included studies in each type-domain combination. The largest concentration of evidence was observed for virtual humanoid agents in health management and health education (n=25), followed by physical humanoid robots in mental health promotion (n=17). Physical humanoid robots were the only type represented across all 3 functional domains, including physiological health promotion (n=6). In contrast, virtual animal-shaped agents were represented by only 1 study, and mechanical robots remained limited overall. Across functional domains, physiological health promotion was the least represented area (6/83, 7.2%), highlighting a clear crosscutting evidence gap in the current literature.

The bubble map shows the distribution of the 83 included studies across embodied intelligence types and functional domains. Bubble size and the number shown within each bubble represent the number of studies in each category combination. Empty cells indicate that no studies were identified for that specific type-domain combination.

### Acceptability and Effectiveness of Embodied Intelligence in Health Care

To strengthen coherence, Table 4 presents a role-based framework mapping the available evidence on acceptability and effectiveness of embodied intelligence across the 3 domains (health management and health education, mental health promotion, and physiological health promotion).

**Table 4.** A role-based framework mapping evidence on the acceptability and effectiveness of embodied intelligence in health care (N=83).

Role category	Studies, n (%)	Key findings on effectiveness	Key findings on acceptability
Health management and health education	40 (48.2)	Demonstrated efficacy in improving patient knowledge, medication adherence, and self-management skills	Generally high acceptance, particularly when systems were perceived as helpful and nonintrusive
Mental health promotion	37 (44.6)	Shown to reduce anxiety and depression symptoms, improve cognitive function, and enhance social interaction in older populations	High acceptance reported, especially for anthropomorphic designs; some studies noted concerns about privacy and overreliance
Physiological health promotion	6 (7.2)	Limited evidence, but promising results in promoting physical activity and assisting with rehabilitation exercises	Mixed acceptance; users valued functionality but sometimes found the technology complex or impersonal

### Evidence of Acceptability

Evidence of acceptability refers to users’ perceptions, satisfaction, usability, engagement, and willingness to adopt embodied intelligence systems.

As summarized in Table 4, about 12 studies examined the acceptability of embodied intelligence in health care applications, predominantly using mixed methods designs. Most focused on health management and education, with fewer addressing mental health promotion; however, heterogeneity in acceptability measures limited direct cross-study comparisons. These findings primarily reflect user experience and perceived feasibility, rather than measured health or behavioral outcomes.

In health management and education, acceptability appears to be shaped by an interplay of human, device-related, and environmental factors. Device-related considerations include anthropomorphic design features and interactive capabilities. Baptista et al [43] reported that acceptability increased when embodied intelligence was framed as a supportive coach rather than an authoritative medical expert in diabetes care. Similarly, Heffner et al [47] found that anthropomorphic appearance and movement fluidity were associated with higher acceptance, underscoring the importance of anthropomorphic design. Consistent with this, Boustani et al [52] and Orejana et al [34] reported that voice interaction and light-based feedback can strengthen social presence and reduce perceived isolation, which was associated with enhanced acceptability. Regarding interactive functions, Reilly et al [36] observed in focus groups that an animal-like robot (Paro) elicited high acceptability by eliciting positive nonverbal responses (eg, petting and smiling), although one participant with animal aversion showed lower engagement. Human and environmental factors were also influential. Zhang [42], Boumans et al [21], and Broadbent et al [23] reported that health literacy, familiarity with technology, education level, and health status were associated with acceptability. Clinical versus home settings also emerged as an important contextual factor shaping acceptability.

Mental health promotion studies showed similar influences of individual and environmental factors on acceptability. Cobo Hurtado et al [68] found that older adults’ initial acceptability of cognitive intervention functions was affected by technophobia; as proficiency increased, the negative influence of anxiety on acceptability diminished. In cross-setting comparisons, Jegundo et al [85] noted that embodied intelligence was more readily accepted in institutional health care settings, whereas home environments required personalized adaptations (eg, functional customization and optimized interaction modes) to enhance acceptability.

### Evidence of Effectiveness

#### Overview

In this review, evidence of effectiveness refers to reported impacts of embodied intelligence interventions on health-related, behavioral, cognitive, or psychological outcomes.

As shown in Table 4, effectiveness evidence was primarily derived from RCTs (22 studies), concentrated in health management and education (12 studies) and mental health promotion (10 studies).

#### Health Management and Education

In this domain, embodied intelligence–based interventions generally reported favorable effects on health behaviors, clinical indicators, and health literacy. Studies by Bickmore et al [51], Robinson and Kavanagh [57], Reilly et al [36], National Clinical Trial [32], and Bickmore et al [50] compared embodied intelligence interventions with traditional management approaches and reported improvements in management goals such as increasing monthly average steps, reducing high-calorie food intake, and weight control. Gardiner et al [53] also reported improvements in stress management and healthy eating behaviors.

Studies by Moyle et al [31], King et al [55], and Jack et al [28] compared embodied intelligence–based health education with traditional health manual–based education and reported better outcomes. Specifically, patients reported higher self-rated quality of life and better adherence to healthy behaviors [54,98], and pregnant women had fewer clinic visits and lower prepregnancy risk ratios [55]. These findings are consistent with the results reported by Gong et al [45].

Bickmore et al [19] compared voice-based embodied intelligence with traditional web-based health information searching and reported higher search success rates and user satisfaction in the embodied intelligence group. In addition, Boumans et al [21] conducted an RCT with 42 older adults and found that embodied intelligence achieved accuracy comparable to health care providers in collecting disease-related information while reporting higher user acceptability.

#### Mental Health Promotion

In this domain, the evidence suggests that embodied intelligence may improve mental health outcomes and provide emotional support. The reported effects can be summarized in 3 dimensions: improvement of psychological symptoms, emotional support, and multimodal intervention.

For the improvement of psychological symptoms, several RCTs reported the benefits of embodied intelligence. For example, Karhiy et al [61] 3-arm trial reported that the embodied intelligence group achieved reductions in perceived stress comparable to human therapists and showed higher compliance than both the human-therapist and chatbot groups. Chen et al [100] and Jøranson et al [81] reported reductions in psychological distress in people with dementia and improvements in agitation and depressive symptoms in older adults. In addition, an RCT by Park et al [66] involving 135 older adults with mild cognitive impairment reported that embodied intelligence–assisted training improved cognitive function and reduced negative emotions.

Regarding emotional support, Robinson et al [82,83] and Lavin et al [73] reported that embodied intelligence can establish emotional connections with older adults and function as a social catalyst, alleviating loneliness and depressive feelings through interaction elements such as touch and conversation.

For multimodal intervention, several studies suggested added value relative to traditional approaches. Sebastian and Richards [91] reported that multimodal interaction (eg, language and expressions) reduced psychological stigmatization more than conventional staff-delivered interventions. Robinson et al [12] reported that mindfulness guidance delivered via embodied intelligence was more effective than traditional audio-guided methods, and Crossman et al [87] reported reduced anxiety in children through multimodal features such as facial expressions, sound, and touch.

Unlike acceptance-related evidence, these studies primarily evaluated intervention effectiveness using objective or validated outcome measures.

### Synthesis of Results

Overall, the charted evidence indicates that embodied intelligence in health care is concentrated in 5 embodiment forms and 3 principal functional domains. The evidence base is most developed for virtual humanoid agents and physical humanoid robots, particularly in health management and health education and in mental health promotion. By contrast, virtual animal-shaped agents, mechanical robots, and applications in physiological health promotion remain comparatively underrepresented.

The evidence gap map further showed that the distribution of evidence across embodiment types and functional domains was uneven. Darker concentrations of evidence were observed for virtual humanoid agents in health management and health education and for physical humanoid robots in mental health promotion, whereas several type-domain combinations contained very limited or no evidence. Across embodiment types, physiological health promotion was consistently the least represented domain, indicating a cross-cutting research gap.

To support an integrative presentation of the findings, Table 4 summarizes the evidence on acceptability and effectiveness across the 3 major functional domains. Taken together, the results suggest that embodied intelligence has been most extensively investigated in aging-related and psychosocial care contexts, that acceptability is generally reported as favorable but context-dependent, and that evidence of effectiveness is promising but concentrated in a limited number of domains and study designs.

## Discussion

### Summary of Evidence

This scoping review systematically synthesizes the research progress and application landscape of embodied intelligence in health care. The literature shows sustained growth in publications and increasingly diversified implementation scenarios. Two recurring development directions emerge across the included studies: anthropomorphism and intelligence. Anthropomorphic design involves attributing human-like physical characteristics, motivations, intentions, and even emotional states to nonhuman entities [12,101]. Empirical work suggests that anthropomorphic features can enhance user experience via multimodal interaction (eg, verbal, facial, and textual modalities) and may improve perceived trust and safety [98].

In parallel, advances in AI—particularly large language models—have expanded the potential for personalized interaction. However, current human-agent interactions remain constrained by limitations in multimodal fusion, natural language understanding, and affective computing, which can affect fluency, accuracy, and recognition of users’ emotions and needs. As shown by ter Stal et al [40], suboptimal personalization mechanisms and inaccurate information can reduce user compliance when embodied agents are used for health management. Taken together, these findings suggest that “more human-like” and “more intelligent” designs are promising but must be evaluated against safety, accuracy, and person-centered interaction quality before large-scale deployment.

Consistent with the thematic mapping, embodied intelligence in health care clusters into 3 functional domains: health management and education, mental health promotion, and physical health promotion. The predominance of studies in health management/education and mental health promotion indicates a current research focus on domains where conversational interaction, engagement, and relational support are central and where embodied agents may plausibly address workforce shortages and access barriers.

For health management and education, embodied intelligence appears advantageous because it can deliver multimodal guidance, provide tailored interaction, and support access independent of time and location. Reported levels of acceptance and compliance indicate feasibility for supporting—rather than uniformly replacing—clinical tasks in management and educational workflows, especially under resource constraints and uneven geographic distribution. In health education, multimodal interaction (voice, text, and facial expressions) has been reported to deliver personalized health knowledge and behavior guidance with efficacy comparable to medical professionals [55], while potentially reducing labor demands. Future research should prioritize clinically safe personalization (eg, accurate information provision, appropriate role framing, and escalation pathways) to ensure that convenience does not compromise care quality.

For mental health promotion, the literature highlights 2 particularly salient mechanisms: psychological intervention and emotional companionship. Regarding psychological intervention, evidence suggests that users—especially those with mental health challenges—may disclose more when interacting with a nonhuman agent than with traditional human-delivered approaches, potentially due to greater perceived privacy protection and reduced psychological defensiveness [102]. Regarding emotional companionship, the potential value is pronounced in older adults, where increasing numbers of seniors living alone and limited companionship resources create an urgent gap. Embodied intelligence may contribute through functional substitution (eg, daily assistance) and social substitution (eg, companionship), with anthropomorphic features increasing social presence and facilitating trust and emotional bonding [103]. Studies also indicate that emotion recognition and responsive interaction may alleviate loneliness and depressive symptoms among older adults [103,104], including those with cognitive decline. Importantly, acceptance and experience vary by setting (eg, nursing homes vs home care) [77]. Given the global shift toward home-based aging [105], future studies should prioritize ecologically valid home deployments, with design choices that fit household routines and caregiver workflows, to strengthen real-world transferability.

By contrast, embodied intelligence remains less represented in physical health promotion, which may reflect higher technical thresholds, stricter safety/access standards, and greater demands for robust performance in physical interaction [106]. Users may accept robot-assisted rehabilitation more readily than conventional training, potentially because gamified interfaces and real-time feedback enhance engagement [107]. Compared with traditional assistive devices, anthropomorphic interaction and emotional intelligence may improve communication and sustained participation [108]. To strengthen evidence, future work should benchmark interventions against relevant standards and usual-care comparators and report outcomes in a way that supports cross-study synthesis.

This review also synthesizes evidence on acceptability and effectiveness. Acceptability varies across studies and appears to be shaped by individual characteristics, robot-related factors, and environmental context. Unlike typical consumer products, acceptance in health care is tied not only to cost but also to perceived health value, trust in the technology, alignment with the agent’s role, and interaction experience, as well as perceived fit and personalization within a specific care scenario [109]. Evidence of effectiveness is currently concentrated in health management/education and mental health promotion. Although only 22 RCTs were identified, all 22 reported positive effects, and some suggested benefits beyond traditional approaches. However, effectiveness assessment remains hindered by no standardized outcomes and heterogeneous methods, limiting comparability and generalizability. Establishing more consistent evaluation criteria would facilitate evidence synthesis. In addition, 2 quasi-experimental studies [97,110] highlight how technical limitations—particularly nonpersonalized or inappropriate feedback—can undermine perceived usability, trust, and acceptance, reinforcing the importance of optimizing interaction quality and safety.

This scoping review is innovative in providing a data-driven map of embodied intelligence in health care across embodiment forms, functional roles, settings, and evidence types. Unlike prior reviews that focus on a single device category or narrow clinical domain, this review integrates both virtual and physical embodiments and synthesizes acceptability and effectiveness evidence within a unified framework. This synthesis clarifies where evidence is most mature (health management and education; mental health promotion) and where major gaps remain (physiological health promotion, standardized outcomes, and longitudinal real-world evaluations). Our findings also highlight recurring determinants of implementation success, including role framing, interaction quality, and contextual fit across settings. In practice, these results can guide stakeholders to match embodiment and function to care needs and settings, while prioritizing safe personalization and ethically grounded deployment. Future research should focus on rigorous, ecologically valid evaluations and standardized reporting to support translation into scalable health care practice.

### Limitations

This study limited its literature inclusion to publications in Chinese and English, which may have introduced selection bias by excluding relevant evidence published in other languages. Publication bias is also a concern, as studies reporting positive outcomes are more likely to be published than those reporting null or negative findings; this may lead to an overrepresentation of RCTs demonstrating the efficacy of embodied intelligence and an underrepresentation of potential limitations. In addition, a substantial proportion of included studies were conducted in laboratory-based evaluation contexts, which may limit ecological validity and the generalizability of findings to real-world care settings. Moreover, consistent with the purpose of a scoping review, we did not conduct a formal critical appraisal of study quality. Therefore, findings—particularly those related to acceptability and effectiveness—should be interpreted cautiously, as the methodological rigor of included studies may vary and could influence the strength of conclusions.

### Conclusions

This scoping review provides a comprehensive synthesis of current evidence on the applications of embodied intelligence in health care, highlighting both emerging trends and critical gaps in the literature. The findings indicate that existing research has predominantly focused on health management and mental health promotion, while evidence for physiological health promotion remains limited. In addition, certain forms of embodied intelligence—particularly virtual animal-shaped agents and mechanical robots—are markedly underrepresented, and studies conducted in real-world clinical settings, especially hospitals, are scarce.

Based on these findings, future research should prioritize addressing these gaps through rigorous and longitudinal study designs, including RCTs and implementation-focused studies conducted in routine health care environments. Such efforts are essential to better understand not only the effectiveness of embodied intelligence interventions but also their sustainability, scalability, and integration into existing care systems. From the perspective of health care and nursing practice, embodied intelligence has the potential to serve as a complementary tool for health education, chronic disease management, and psychosocial support. However, successful adoption will depend on careful attention to usability, workflow integration, and ethical considerations, as well as on clearly defining the roles of nurses and other health care professionals as facilitators, supervisors, and mediators of human-technology interaction. Addressing these practical considerations alongside methodological gaps will be crucial for translating embodied intelligence from experimental settings into meaningful, patient-centered health care practice.

## Supplementary material

10.2196/83871 Multimedia Appendix 1 Search strategy.

10.2196/83871 Multimedia Appendix 2 Characteristics of all included studies.

10.2196/83871 Checklist 1 PRISMA-ScR checklist.

10.2196/83871 Checklist 2 PRISMA-S checklist.

## Abbreviations

AI: artificial intelligence
CNKI: China National Knowledge Infrastructure
CQVIP: VIP Chinese Journal Database
OSF: Open Science Framework
PCC: population, concept, and context framework
PRESS: Peer Review of Electronic Search Strategies
PRISMA-S: an extension to the PRISMA statement for reporting literature searches in systematic reviews
PRISMA-ScR: Preferred Reporting Items for Systematic Reviews and Meta-Analyses Extension for Scoping Reviews
RCT: randomized controlled trial
