# Supplementary material for: Embodied Intelligence Applications in Health Care Populations: Scoping Review
Source: J Med Internet Res. 2026 Jun 12;28:e83871. doi: 10.2196/83871 (PMC13262780; doi:10.2196/83871)
Supplement: Multimedia Appendix 1 [file jmir-v28-e83871-s001.docx]

Search Strategy

| PubMed | `"Artificial Intelligence"[Mesh] OR "Robotics"[Mesh] OR "Humanoid Robots"[Mesh] OR "Social Robots"[Title/Abstract] OR "socially assistive robot*"[Title/Abstract] OR "companion robot*"[Title/Abstract] OR "care robot*"[Title/Abstract] OR "healthcare robot*"[Title/Abstract] OR "medical robot*"[Title/Abstract] OR "humanoid robot*"[Title/Abstract] OR robot*[Title/Abstract] OR robotics[Title/Abstract] OR "virtual agent*"[Title/Abstract] OR "conversational agent*"[Title/Abstract] OR "embodied conversational agent*"[Title/Abstract] OR ECA[Title/Abstract] OR ECAs[Title/Abstract] OR "relational agent*"[Title/Abstract] OR avatar*[Title/Abstract] OR "virtual human*"[Title/Abstract] OR "virtual coach*"[Title/Abstract] OR "embodied intelligence"[Title/Abstract] OR "embodied AI"[Title/Abstract] OR "embodied artificial intelligence"[Title/Abstract] OR "embodied agent*"[Title/Abstract] OR "artificial embodied agent*"[Title/Abstract])  AND ( "Delivery of Health Care"[Mesh] OR "Patient Care"[Mesh] OR "Home Care Services"[Mesh] OR "Long-Term Care"[Mesh] OR "Hospitals"[Mesh] OR "Rehabilitation"[Mesh] OR "Mental Health Services"[Mesh] OR health*[Title/Abstract] OR healthcare[Title/Abstract] OR "health care"[Title/Abstract] OR medical[Title/Abstract] OR clinical[Title/Abstract] OR patient*[Title/Abstract] OR care[Title/Abstract] OR nursing[Title/Abstract] OR hospital*[Title/Abstract] OR rehabilitation[Title/Abstract] OR therap*[Title/Abstract] OR "mental health"[Title/Abstract] OR "home care"[Title/Abstract] OR "care home*"[Title/Abstract] OR "nursing home*"[Title/Abstract] OR "long-term care"[Title/Abstract] OR "aged care"[Title/Abstract] OR "elder care"[Title/Abstract])` |
| --- | --- |
| Web of Science | `( TS=(("artificial intelligence" OR robotics OR robot* OR "humanoid robot*" OR "social robot*" OR "socially assistive robot*" OR "companion robot*" OR "care robot*" OR "healthcare robot*" OR "medical robot*" OR "virtual agent*" OR "conversational agent*" OR "embodied conversational agent*" OR ECA OR ECAs OR "relational agent*" OR avatar* OR "virtual human*" OR "virtual coach*" OR "embodied intelligence" OR "embodied AI" OR "embodied artificial intelligence" OR "embodied agent*" OR "artificial embodied agent*") AND ( health* OR healthcare OR "health care" OR medical OR clinical OR patient* OR nursing OR hospital* OR rehabilitation OR therap* OR "mental health" OR "home care" OR "care home*" OR "nursing home*" OR "long-term care" OR "aged care" OR "elder care" )))` |
| Cochrane Library | `(MeSH descriptor: [Artificial Intelligence] explode all trees OR MeSH descriptor: [Robotics] explode all trees OR robot*:ti,ab,kw OR robotics:ti,ab,kw OR "humanoid robot*":ti,ab,kw OR "social robot*":ti,ab,kw OR "socially assistive robot*":ti,ab,kw OR "companion robot*":ti,ab,kw OR "care robot*":ti,ab,kw OR "healthcare robot*":ti,ab,kw OR "medical robot*":ti,ab,kw OR "virtual agent*":ti,ab,kw OR "conversational agent*":ti,ab,kw OR "embodied conversational agent*":ti,ab,kw OR ECA:ti,ab,kw OR ECAs:ti,ab,kw OR "relational agent*":ti,ab,kw OR avatar*:ti,ab,kw OR "virtual human*":ti,ab,kw OR "virtual coach*":ti,ab,kw OR "embodied intelligence":ti,ab,kw OR "embodied AI":ti,ab,kw OR "embodied artificial intelligence":ti,ab,kw OR "embodied agent*":ti,ab,kw OR "artificial embodied agent*":ti,ab,kw) AND ( MeSH descriptor: [Delivery of Health Care] explode all trees OR MeSH descriptor: [Patient Care] explode all trees OR MeSH descriptor: [Home Care Services] explode all trees OR MeSH descriptor: [Long-Term Care] explode all trees OR MeSH descriptor: [Hospitals] explode all trees OR MeSH descriptor: [Rehabilitation] explode all trees OR health*:ti,ab,kw OR healthcare:ti,ab,kw OR "health care":ti,ab,kw OR medical:ti,ab,kw OR clinical:ti,ab,kw OR patient*:ti,ab,kw OR nursing:ti,ab,kw OR hospital*:ti,ab,kw OR rehabilitation:ti,ab,kw OR therap*:ti,ab,kw OR "mental health":ti,ab,kw OR "home care":ti,ab,kw OR "care home*":ti,ab,kw OR "nursing home*":ti,ab,kw OR "long-term care":ti,ab,kw OR "aged care":ti,ab,kw OR "elder care":ti,ab,kw)` |
| MEDLINE | `(1. exp Artificial Intelligence/  2. exp Robotics/  3. robot*.ti,ab,kf.  4. robotics.ti,ab,kf.  5. humanoid robot*.ti,ab,kf.  6. social robot*.ti,ab,kf.  7. socially assistive robot*.ti,ab,kf.  8. companion robot*.ti,ab,kf.  9. care robot*.ti,ab,kf.  10. healthcare robot*.ti,ab,kf.  11. medical robot*.ti,ab,kf.  12. virtual agent*.ti,ab,kf.  13. conversational agent*.ti,ab,kf.  14. embodied conversational agent*.ti,ab,kf.  15. ECA.ti,ab,kf.  16. ECAs.ti,ab,kf.  17. relational agent*.ti,ab,kf.  18. avatar*.ti,ab,kf.  19. virtual human*.ti,ab,kf.  20. virtual coach*.ti,ab,kf.  21. embodied intelligence.ti,ab,kf.  22. embodied AI.ti,ab,kf.  23. embodied artificial intelligence.ti,ab,kf.  24. embodied agent*.ti,ab,kf.  25. artificial embodied agent*.ti,ab,kf.  26. or/1-25  27. exp Delivery of Health Care/  28. exp Patient Care/  29. exp Home Care Services/  30. exp Long-Term Care/  31. exp Hospitals/  32. exp Rehabilitation/  33. exp Mental Health Services/  34. health*.ti,ab,kf.  35. healthcare.ti,ab,kf.  36. health care.ti,ab,kf.  37. medical.ti,ab,kf.  38. clinical.ti,ab,kf.  39. patient*.ti,ab,kf.  40. care.ti,ab,kf.  41. nursing.ti,ab,kf.  42. hospital*.ti,ab,kf.  43. rehabilitation.ti,ab,kf.  44. therap*.ti,ab,kf.  45. mental health.ti,ab,kf.  46. home care.ti,ab,kf.  47. care home*.ti,ab,kf.  48. nursing home*.ti,ab,kf.  49. long-term care.ti,ab,kf.  50. aged care.ti,ab,kf.  51. elder care.ti,ab,kf.  52. or/27-51  53. 26 and 52` |
| APA PsycNet | `(AB("artificial intelligence" OR robotics OR robot* OR "humanoid robot*" OR "social robot*" OR "socially assistive robot*" OR "companion robot*" OR "care robot*" OR "virtual agent*" OR "conversational agent*" OR "embodied conversational agent*" OR ECA OR ECAs OR "relational agent*" OR avatar* OR "virtual human*" OR "virtual coach*" OR "embodied intelligence" OR "embodied AI" OR "embodied artificial intelligence" OR "embodied agent*" OR "artificial embodied agent*") OR TI("artificial intelligence" OR robotics OR robot* OR "humanoid robot*" OR "social robot*" OR "socially assistive robot*" OR "companion robot*" OR "care robot*" OR "virtual agent*" OR "conversational agent*" OR "embodied conversational agent*" OR ECA OR ECAs OR "relational agent*" OR avatar* OR "virtual human*" OR "virtual coach*" OR "embodied intelligence" OR "embodied AI" OR "embodied artificial intelligence" OR "embodied agent*" OR "artificial embodied agent*"))  AND ( AB(health* OR healthcare OR "health care" OR medical OR clinical OR patient* OR nursing OR hospital* OR rehabilitation OR therap* OR "mental health" OR "home care" OR "care home*" OR "nursing home*" OR "long-term care" OR "aged care" OR "elder care")OR  TI(health* OR healthcare OR "health care" OR medical OR clinical OR patient* OR nursing OR hospital* OR rehabilitation OR therap* OR "mental health" OR "home care" OR "care home*" OR "nursing home*" OR "long-term care" OR "aged care" OR "elder care"))` |
| CQVIP | `(主题 = ("具身智能" OR "具身人工智能" OR "具身对话代理" OR "具身会话代理" OR "具身智能体" OR "人工具身代理" OR "虚拟代理" OR "虚拟智能体" OR "对话代理" OR "对话智能体" OR "关系代理" OR "虚拟人" OR "虚拟数字人" OR "头像代理" OR "机器人" OR "机器人技术" OR "类人机器人" OR "社交机器人" OR "陪伴机器人" OR "辅助机器人" OR "照护机器人" OR "医疗机器人"))  AND  (主题 = ("医疗" OR "医疗保健" OR "健康照护" OR "健康护理" OR "卫生保健" OR "临床" OR "患者" OR "护理" OR "医院" OR "康复" OR "治疗" OR "心理健康" OR "居家照护" OR "家庭照护" OR "养老" OR "长期照护" OR "护理院" OR "养老院"))` |
| China National Knowledge Infrastructure (CNKI) | `( SU = ("具身智能" OR "具身人工智能" OR "具身对话代理" OR "具身会话代理" OR "具身智能体" OR "人工具身代理" OR "虚拟代理" OR "虚拟智能体" OR "对话代理" OR "对话智能体" OR "关系代理" OR "虚拟人" OR "虚拟数字人" OR "头像代理" OR "机器人" OR "机器人技术" OR "类人机器人" OR "社交机器人" OR "陪伴机器人" OR "辅助机器人" OR "照护机器人" OR "医疗机器人"))  AND  ( SU = ("医疗" OR "医疗保健" OR "健康照护" OR "健康护理" OR "卫生保健" OR "临床" OR "患者" OR "护理" OR "医院" OR "康复" OR "治疗" OR "心理健康" OR "居家照护" OR "家庭照护" OR "养老" OR "长期照护" OR "护理院" OR "养老院"))` |
