# Supplementary material for: Embodied Intelligence Applications in Health Care Populations: Scoping Review
Source: J Med Internet Res. 2026 Jun 12;28:e83871. doi: 10.2196/83871 (PMC13262780; doi:10.2196/83871)
Supplement: Multimedia Appendix 2 [file jmir-v28-e83871-s002.docx]

**Study Characteristics (N=83)**

| **No.** | **Author， Year** | **Country** | **Study Design** | **Study Subject** | **Health care delivery** | **Role (1=Health management and health education ；2=Mental health promotion. ；3=Physiological health promotion)** | Type of Embodied Intelligence (1=ECA(humanoid)；2=ECA(animal-shaped)；3=Humanoid Robot；4=animal robot;5=assistive robots/mechanical robot) | Name of Embodied Intelligence | Application Scenarios：(**1=lab；2=care home；3=home；4=hospital)** | **Key Outcomes** |
| --- | --- | --- | --- | --- | --- | --- | --- | --- | --- | --- |
| 1 | Guojing Ma et al., 2023 [58] | China | Quasi-experiments | Esophageal cancer patients | Health education | 1 | 5 | Intelligent service robot | 4 | Enhance patients' full comprehension of health education, achieve high satisfaction with educational provision, and reduce the time required for health education relative to nursing duties. |
| 2 | Robinson et al., 2023 [12] | Australia | Randomized controlled trials | Undergraduate | Mental health promotion (mindfulness training) | 2 | 3 | Humanoid Robot（Pepper） | 1 | Promote participants' sense of well-being and enhance their receptiveness to mindfulness. |
| 3 | Boumans et al., 2020 [22] | Netherlands | Cross-over trals | Older adults | Patient-reported outcomes/case collection | 1 | 3 | Humanoid Robot（Pepper） | 1 | Social robots can effectively and efficiently collect PROM data from elderly individuals. The recorded data demonstrated a reliability rate of 99.6%. |
| 4 | Wainer et al., 2014 [89] | UK | Pilot study | Autism spectrum disorder | Psychotherapy | 2 | 3 | Humanoid Robot(KASPAR) | 1 | Findings revealed that children found the activity more engaging, appeared more immersed in play, and exhibited improved cooperative behaviour with their peers. |
| 5 | Bott et al., 2019 [67] | USA | Pre-post study | Older adults | Elderly care | 2 | 2 | Embodied conversational agent | 4 | Delirium occurred less frequently (P<.001), fewer reports of loneliness were noted (P=.01), and fewer falls were recorded. No significant differences were observed in self-reported depressive symptoms. |
| 6 | Bickmore et al., 2013 [44] | USA | Randomized controlled trials | Older adults | Exercise guidance | 1 | 1 | Embodied conversational agent（Gabby system） | 4 | Increase the walking rate among the elderly. |
| 7 | Robinson et al., 2021 [57] | Australia | Randomized controlled trials | Public | Health education(diet guidance) | 1 | 3 | Humanoid robot(NAO) | 1 | Improve patients' health behaviours. |
| 8 | Louie et al., 2021 [94] | USA | Case studies | Autism spectrum disorder | Disease treatment-listening comprehension intervention | 3 | 3 | Humanoid robot(NAO) | 1 | Enhance the child's auditory comprehension skills. Children with ASD often require tailored intervention programmes due to their varying abilities and preferences. |
| 9 | Baptista et al., 2020 [43] | Australia | Mixed-method studies | Diabetic patients | Health management | 1 | 1 | Embodied conversational agent(Laura) | 3 | ECA is an acceptable method for delivering T2D self-management education and support. A humanoid character providing ongoing, friendly, non-judgmental, emotionally supportive and motivational assistance has proved highly popular. |
| 10 | Nkabane-Nkholongo et al., 2023 [48] | South Africa | Qualitative study | Women | Health management | 1 | 1 | Embodied conversational agent（Gabby system） | 3 | Nthabi's clinically tailored, culturally sensitive and trustworthy content has the potential to improve access to sexual and reproductive health information for young women in low- and middle-income countries such as Lesotho. |
| 11 | Shidara et al., 2024 [62] | Japan | Quasi-experiments | Members of the public with psychological issues | Psychotherapy(CBT) | 2 | 1 | Embodied conversational agent(Greta) | 3 | Adjusting the number of questions based on perceived distress levels can enhance the CBT outcomes delivered by ECA. |
| 12 | Görer et al., 2017 [46] | Turkey | Quasi-experiments | Older adults | Exercise guidance | 1 | 3 | Humanoid Robot(NAO) | 2 | Older adults can complete exercises with the assistance of robots, but improved machine feedback can deliver a better user experience for them. |
| 13 | Heffner et al., 2021 [47] | USA | Pilot study | Sexual minorities | Quit smoking | 1 | 1 | Embodied conversational agent（EQQUAL） | 3 | The smoking cessation rate is three times that of other digital programmes specifically targeting sexual and gender minority youth to date, and six to thirteen times that of non-targeted digital smoking interventions for sexual and gender minority youth. |
| 14 | Suganuma et al., 2018 [63] | Japan | Pilot study | Public | Psychotherapy(CBT) | 2 | 1 | Embodied conversational agent（SABORI） | 3 | Internet-based cognitive behavioural therapy incorporating embedded conversational agents may be employed in mental health care. |
| 15 | ter Stal et al., 2021 [40] | Netherlands | Quasi-experiments | COPD patients | Health management | 1 | 1 | Embodied conversational agent（Sylvia） | 4 | The likelihood of patients adhering to medical advice is reduced. |
| 16 | Reilly et al., 2023 [36] | USA | Randomized controlled trials | Veterans | Chronic pain management | 1 | 1 | Embodied conversational agent（VACT-CP system） | 1 | VACT-CP was feasible and usable. It led to a higher proportion of preconception risks reaching the action/maintenance stage of change compared to a control letter. |
| 17 | Martínez-Miranda et al., 2019 [30] | Mexico | Pilot study | Individuals with suicidal tendencies | Suicide prevention and monitoring | 1 | 1 | Embodied conversational agent（HelPath） | 1 | Good user compliance. |
| 18 | Bickmore et al., 2013 [51] | USA | Pilot study | Public | Health education(diet exercise guidance) | 1 | 1 | Embodied conversational agent（Gabby system） | 3 | Significantly effective in changing health behaviours. |
| 19 | Beinema et al., 2022 [49] | Netherlands | Quasi-experiments | Public | Health education | 1 | 1 | Embodied conversational agent（Olivia） | 3 | Personalisation is crucial during interactions: tailoring coaching dialogues with the ECA by having Coach automatically suggest topics tailored to the user is regarded as a natural evolution within the interaction process. |
| 20 | Broadbent et al., 2016 [24] | New Zealand | Mixed-method studies | Older adults | Elderly care | 1 | 1 | Health-care robots(Guide and Cafero) | 3 | The intervention group perceived the robots as possessing greater agency and experience than the control group. The perceived agency of robots in both groups declined over time. |
| 21 | Karhiy et al., 2024 [61] | New Zealand | Randomized controlled trials | Undergraduate | Mental health promotion (mindfulness training) | 2 | 1 | Embodied conversational agent/virtual human | 3 | All three approaches proved effective, with research findings supporting the use of virtual avatars for mindfulness interventions among stressed students. Compared to remote therapy and chatbots, virtual avatars may offer the advantage of enhancing adherence among student populations. |
| 22 | Sebastian and Richards, 2017 [92] | Australia | Randomized controlled trials | Undergraduate | Mental health promotion （reducing self-stigma in eating disorders） | 2 | 1 | Embodied conversational agent（Sarah and Alice） | 3 | Exposure and educational intervention strategies delivered through ECA can effectively reduce stigma surrounding eating disorders among university students. |
| 23 | O’Leary et al., 2023 [91] | USA | Mixed-method studies | Adult patients with depression | Mental health promotion （reducing self-stigmatisation in depression） | 2 | 1 | Embodied conversational agent（Clara） | 1 | The ECA-based intervention was found to be feasible and acceptable. It facilitated positive shifts in attitudes toward mental health and primed care-seeking behaviors. |
| 24 | Hun Lee et al., 2023 [97] | Singapore | Pilot study | Stroke patients (due to COVID-19, the subjects actually recruited were healthy individuals) | Rehabilitation training | 3 | 3 | Humanoid robot(NAO) | 1 | Adapt to new users and achieve improved performance. |
| 25 | Law et al., 2019 [29] | New Zealand | Qualitative study | Older adults with mild cognitive impairment | Elderly care | 1 | 3 | Assistive robots(Silbot robot) | 2 | Assistive robots are considered beneficial, particularly for reminders and safety checks, with the potential to offer companionship, reassurance and alleviate the burden on carers. |
| 26 | Cobo Hurtado et al., 2021 [68] | Spain | Pilot study | Older adults | Elderly care | 2 | 3 | Social robots/humanoid Robot（Pepper） | 2 | Robots can assist users in maintaining physical and mental activity while fostering an emotional connection between users and the robot. Furthermore, user acceptance is generally favourable. |
| 27 | Boustani et al., 2021 [52] | USA | Quasi-experiments | Alcohol abusers | Health education | 1 | 1 | Embodied conversational agent/empathic embodied virtual agent (eEVA) system） | 3 | Participants reported high acceptability and practicality of the technology, whose performance enabled it to successfully deliver brief behavioural health interventions via the internet to reduce alcohol use. |
| 28 | Lane et al., 2016 [72] | USA | Pilot study | Older adults | Elderly care | 2 | 4 | Social robot(Paro） | 2 | Paro may represent an effective non-pharmacological approach for managing dementia-related emotional and behavioural issues among veterans in VA long-term care facilities. They also observed that Paro is most suitable for presentation to relatively calm and approachable residents, rather than those actively exhibiting behavioural or emotional problems. |
| 29 | Chen et al., 2020 [100] | 中国香港 | Randomized controlled trials | Dementia patients | Elderly care | 2 | 3 | Humanoid robot(Kabochan) | 2 | Kabochan may effectively alleviate short-term neuropsychiatric symptoms in dementia residents and reduce the distress experienced by associated carers. |
| 30 | Jøranson et al., 2015 [81] | Norway | Randomized controlled trials | Dementia patients | Elderly care | 2 | 4 | Social robot(Paro） | 2 | Alleviate depression and agitation. |
| 31 | Khosla et al., 2013 [71] | Australia | Pilot study | Older adults | Elderly care(Emotional companionship) | 2 | 5 | Social assistive communication (service and companion) robot(Matilda) | 2 | The robot showed high interaction levels and was a promising tool for cognitive stimulation. The systematic video evaluation was also found to be effective for assessing user-robot interaction. |
| 32 | Rossi et al., 2020 [88] | Italy | Quasi-experiments | Children | Childcare (reducing vaccine-related anxiety) | 2 | 3 | Social assistive Robots/ humanoid robot(NAO) | 4 | Robotic distraction strategies can reduce fear and anxiety while enhancing well-being across diverse scenarios. |
| 33 | Gardiner et al., 2017 [53] | USA | Randomized controlled trials | Women | Health management | 1 | 1 | Embodied conversational agent（Gabby system） | 3 | ECA technology can assist in enhancing lifestyle information education for women in diverse urban settings, encompassing physical activity, healthy eating, mindfulness, and stress management techniques. |
| 34 | Kitt et al., 2021 [90] | USA | Quasi-experiments | Children | Mental health care | 2 | 4 | Social robot(paro） | 1 | No evidence was found for the anticipated benefits of SAR in alleviating stress in children. |
| 35 | Moyle et al., 2013 [31] | Australia | Randomized controlled trials | Dementia patients | Elderly care | 1 | 4 | Social robot(Paro） | 2 | PARO has a moderate to large positive impact on participants' quality of life. |
| 36 | Leung et al., 2020 [86] | 中国香港 | Case studies | Older adults | Elderly care | 2 | 3 | Humanoid social robots（Ka Ka） | 3 | Providing emotional support for elderly individuals living alone, diversifying the daily activities of older adults, and strengthening the dyadic relationship between older adults and their family carers. |
| 37 | Jøranson et al., 2016 [82] | Norway | Mixed-method studies | Older adults | Elderly care | 2 | 4 | Social robot(Paro） | 2 | Paro appears to serve as an intermediary for enhancing social interaction and fostering engagement. |
| 38 | Robinson et al., 2016 [84] | New Zealand | Randomized controlled trials | Older adults | Elderly care | 2 | 4 | Social robot(Paro） | 3 | Paro offers psychosocial benefits. |
| 39 | Orejana et al., 2015 [34] | New Zealand | Case studies | Rural elderly people | Elderly care | 1 | 5 | Social assistive robots(iRobi) | 4 | In the presence of the robot, visits to primary care and telephone consultations with doctors decreased, alongside an observed improvement in quality of life. Despite encountering technical issues, patients were largely positive, accepting the robot and acknowledging its benefits as a companion. |
| 40 | Pu et al., 2020 [75] | Australia | Qualitative study | Patients with dementia and chronic pain | Chronic pain management | 2 | 4 | Social robot(Paro） | 2 | Alleviate pain. |
| 41 | Lavin et al., 2022 [73] | Canada | Randomized controlled trials | Older adults | Elderly care | 2 | 3 | Humanoid social robots（Grace） | 2 | Reducing loneliness, anxiety, depression and other improved mental health outcomes among older adults, alongside decreased healthcare utilisation and enhanced overall quality of life. |
| 42 | Bickmore et al., 2016 [19] | USA | Randomized controlled trials | Public | Health management (health consultation) | 1 | 1 | Embodied conversational agent（Gabby system） | 1 | When comparing dialogue agent-based search user interfaces with traditional keyword- and facet-based search engine interfaces, participants expressed greater satisfaction with the agents. |
| 43 | Richards et al., 2018 [37] | Australia | Quasi-experiments | Family members of children with urinary incontinence | Health consultation and health guidance | 1 | 1 | Embodied conversational agent（Dr Evie） | 4 | 74 per cent of paediatric patients experienced improvement in urinary incontinence symptoms. Improve treatment adherence. |
| 44 | Jack et al., 2020 [28] | USA | Randomized controlled trials | Pregnant woman | Health management | 1 | 1 | Embodied conversational agent（Gabby system） | 3 | The Gabby system significantly increased the proportion of preconception health risks reaching the action or maintenance stage of change compared to the control group, and this effect was sustained at 12 months. |
| 45 | Demange et al., 2019 [27] | France | Mixed-method studies | Dementia patients | Pain management | 1 | 4 | Social robot(Paro） | 2 | Participants perceived better usability. |
| 46 | D’Onofrio et al., 2019 [79] | Italy | Pilot study | Dementia patients | Elderly care | 2 | 3 | Service robots(MARIO) | 4 | Alleviate depression and loneliness, enhance social connections, resilience and overall quality of life for individuals with dementia. |
| 47 | Zhang et al., 2020 [42] | USA | Quasi-experiments | Public | Health management | 1 | 1 | Embodied conversational agent（Gabby system） | 3 | Participants found the virtual decision coach more effective than standard educational brochures. They were highly engaged in the decision-making process and expressed high satisfaction with the virtual coach's performance. |
| 48 | Ruiz-del-Solar et al., 2021 [76] | Chile | Quasi-experiments | Patients in the isolation ward | Mental health nursing | 2 | 5 | social robot(Pudu) | 4 | Robots can effectively provide mental health care for isolated COVID-19 patients; they can also generate positive emotional experiences for healthcare workers. |
| 49 | Gong et al., 2020 [45] | Australia | Randomized controlled trials | Diabetic patients | Self-management of illness | 1 | 1 | Embodied conversational agent(Laura) | 3 | Enhance quality of life. |
| 50 | Jegundo et al., 2020 [85] | Portugal | Mixed-method studies | Older adults | Elderly care | 2 | 1 | Embodied conversational agent(CaMeLi) | 2 | Acceptance has been positive. |
| 51 | Blindheim et al., 2023 [20] | Norway | Pilot study | Older adults | Elderly care | 1 | 3 | Social robots/humanoid robot（Pepper） | 2 | Participants reported that they enjoyed interacting with social robots. |
| 52 | Chan et al., 2010 [64] | Canada | Quasi-experiments | Dementia patients | Elderly care | 2 | 3 | Human-like socially assistive robot(Brian 2.0) | 1 | Brian 2.0's social interaction capabilities prove effective in engaging individuals in cognitively stimulating games. |
| 53 | Pérez-Zuñiga et al., 2024 [74] | Peru | Pilot study | Undergraduate | Psychotherapy | 2 | 3 | Humanoid robot(Qhali) | 1 | Emotional wellbeing showed improvement, and their perception of psychological interventions using humanoid robots yielded positive outcomes. |
| 54 | Boumans et al., 2019 [21] | Netherlands | Randomized controlled trials | Older adults | Health management | 1 | 3 | Social robots/humanoid robot（Pepper） | 4 | Interactive robots can effectively and acceptably assist healthcare personnel by interviewing elderly individuals. |
| 55 | Hudson et al., 2020 [80] | USA | Qualitative study | Older adults | Elderly care | 2 | 4 | Robotic pet | 2 | Alleviate loneliness among the elderly. |
| 56 | Birks et al., 2016 [78] | Australia | Qualitative study | Older adults | Elderly care | 2 | 4 | Social robot(Paro） | 2 | The use of Paro as a therapeutic tool has demonstrated improvements in residents' emotional states, a reduction in challenging behaviours, and enhanced social interaction. |
| 57 | Broadbent et al., 2014 [23] | New Zealand | Cross-over trals | Older adults | Elderly care | 1 | 5 | Social robots( IrobiQ and Cafero) | 2 | Robots have been found to be acceptable and viable. |
| 58 | Fan et al., 2023 [65] | USA | 3×3 factorial design | Older adults | Elderly care | 2 | 3 | Humanoid robot(NAO ) | 2 | Promoting activity participation and social interaction among older adults. |
| 59 | Yoshii et al., 2023 [41] | Japan | Pilot study | Older adults | Health management(disease screening) | 1 | 3 | Social robots/humanoid robot（Pepper） | 4 | The potential for early and straightforward screening of patients with mild cognitive impairment using prosodic and acoustic features from daily conversations with humanoid robots, with accuracy comparable to that of psychoneuropsychological assessments (the Mini-Mental State Examination). |
| 60 | Fields et al., 2021 [69] | USA | Pilot study | Older adults | Elderly care | 2 | 3 | Humanoid robot(NAO) | 2 |  |
| 61 | Casas et al., 2018 [96] | USA | Pilot study | Cardiac rehabilitation patients | Supportive rehabilitation services | 3 | 3 | Humanoid robot(NAO) | 1 | In this study, the robot condition showed improved patient compliance (shorter response time to requests) compared to the no-robot condition, and all patients reported high motivation. |
| 62 | Chen et al., 2021 [25] | 中国台湾 | Quasi-experiments | Older adults | Health assessment (attention state evaluation) | 1 | 3 | Humanoid robot(RoBoHoN) | 1 | Demonstrated the potential for social companion robots to construct age-related cognitive profiles and provide guidance for attention assessments for elderly individuals in the home. |
| 63 | Kramer et al., 2021 [56] | Netherlands | Quasi-experiments | Older adults | Health education (dietary behaviour) | 1 | 1 | Embodied conversational agent（Sylvia） | 3 | ECAs hold potential for improving healthy dietary behaviours among older adults. |
| 64 | Robins et al., 2014 [95] | UK | Quasi-experiments | Autism spectrum disorder | Treatment of disease | 3 | 3 | Humanoid robot(KASPAR) | 1 | Through tactile interaction with robots, children with autism can learn fundamental cause-and-effect relationships, such as eliciting different responses by touching various parts of the robot. Furthermore, some children are able to respond to the robot's expressions of “sadness” and “happiness” and adjust their own behaviour accordingly. |
| 65 | Curumsing et al., 2024 [26] | Australia | Mixed-method studies | Caregivers of individuals with dementia | Health education | 1 | 1 | Embodied conversational agent（TED） | 1 | The carer formed an emotional bond with the ECA and retained the learning gained from interactions with Ted even after eight weeks. |
| 66 | King et al., 2017 [55] | USA | Randomized controlled trials | Adults aged 50 or above | Health education (physical exercise) | 1 | 1 | Embodied conversational agent（Carmen） | 1 | Influencing key health behaviours among underserved populations to reduce health inequalities. |
| 67 | Pu et al., 2021 [35] | Australia | Pilot study | Dementia patients with chronic pain | Chronic pain management | 1 | 4 | Social robot(Paro） | 2 | PARO can improve sleep patterns in care home residents suffering from dementia and chronic pain. |
| 68 | Nomura et al., 2021 [98] | Japan | Pilot study | Older adults | Elderly care | 3 | 3 | Social robots(Robovie-R3) | 2 | Participants preferred walking alongside the robot rather than walking alone. |
| 69 | Tanioka et al., 2021 [77] | Japan | Qualitative study | Older adults | Elderly care | 2 | 3 | Social robot(Pepper robot and Kabochan) | 2 | Interacting with robots helps elderly individuals with schizophrenia and dementia express joy. |
| 70 | Park et al., 2021 [66] | Korea | Randomized controlled trials | Older adults with mild cognitive impairment | Elderly care (cognitive training) | 2 | 3 | Humanoid robot(Sil-Bot) | 2 | Cognitive training programmes can enhance overall cognitive function and reduce depression during cognitive tasks in elderly individuals with mild cognitive impairment (MCI), which is associated with improvements in memory and executive function. |
| 71 | Crossman et al., 2018 [87] | USA | Randomized controlled trials | Children | Mental health promotion | 2 | 4 | Social robot(Paro） | 1 | SARs may confer benefits to children's mental wellbeing by enhancing positive emotions. |
| 72 | Robinson et al., 2013 [83] | New Zealand | Randomized controlled trials | Older adults | Elderly care | 2 | 4 | Social robot(Paro） | 2 | Paro may reduce feelings of loneliness. |
| 73 | Stara et al., 2021 [39] | Italy | Mixed-method studies | Dementia patients | Elderly care | 1 | 1 | Embodied conversational agent（Anne ） | 2 | Participants rated the agent as easy to use and quick to learn. They felt confident using the system and expressed willingness to use it frequently. Furthermore, 21 out of 55 patients viewed the virtual agent as a friend and assistant they could feel close to, who would remind them of important matters. Conclusion: Technology-based interventions require significant effort, such as personalised features and patient-centred care pathways, to be effective. |
| 74 | Huisman et al., 2019 [70] | Netherlands | Mixed-method studies | Older adults | Elderly care | 2 | 3 | Humanoid robot(Nao) | 2 | All care facilities remain willing to continue using the robot as an alternative means of providing entertainment and rehabilitation programmes for their clients. |
| 75 | Stara et al., 2021 [38] | Italy | Mixed-method studies | Dementia patients | Elderly care | 1 | 1 | Embodied conversational agent（Anne ） | 2 | Participants engaged effectively with the system, perceiving the virtual agent as a companion capable of supporting their memory and fulfilling their enjoyment needs. |
| 76 | Bickmore et al., 2010 [50] | USA | Randomized controlled trials | Patients | Health education | 1 | 1 | Embodied conversational agent（Louise） | 4 | Patients across different levels of health literacy found these agents acceptable for automated health communication interventions and easy to use. |
| 77 | Nct et al., 2020 [32] | Netherlands | Randomized controlled trials | Older adults | Elderly care | 1 | 1 | Embodied conversational agent(PACO) | 3 | The research did not provide any conclusive evidence regarding factors associated with ECA use or health effects. |
| 78 | Hurmuz et al., 2020 [54] | Netherlands | Observational Cohort Study | Individuals aged 55 years or older, and adults aged 18 years or older with type 2 diabetes or chronic pain | Health education | 1 | 1 | Embodied conversational agent(COUCH) | 3 | To determine how long the target group is willing to engage with the virtual coaching system, and whether the virtual coaching system can lead to behavioural change. |
| 79 | Costa et al., 2015 [93] | UK | Qualitative study | Autism spectrum disorder | Treatment of disease | 3 | 3 | Humanoid Robot(KASPAR) | 1 | KASPAR can serve as an effective tool for promoting children's recognition of body parts and as an object for shared attention, thereby enhancing social interactions with human companions. |
| 80 | Olde Keizer et al., 2019 [33] | Netherlands | Mixed-method studies | Older adults | Elderly care | 1 | 3 | Humanoid robot(NAO) | 1 | Social robots hold potential for monitoring and training the health of frail elderly individuals, yet several key usability challenges must first be overcome. |
| 81 | Hudlicka et al., 2013 [60] | USA | Pilot study | Undergraduate | Mental health promotion (mindfulness training) | 2 | 1 | embodied conversational agent（Chris ） | 1 | Mindfulness training facilitated by virtual coaches is not only feasible but may prove more effective than self-directed programmes. |
| 82 | Cho et al., 2024 [59] | South Korea | Quasi-experimental research design (one-group time series & pretest-post test) | Older adults | Emotional companionship | 2 | 3 | Social robot(Pepper） | 2 | Service stability and usefulness have been verified. |
| 83 | Rodriguez-Dominguez et al., 2024 [99] | Spain | Descriptive observational pilot study | Older adults with mild to moderate cognitive impairment | Cognitive training | 2 | 3 | EBO robot | 2 | System video assessment has also demonstrated effectiveness in evaluating human-computer interaction. |
